# Hyoid bone position as an indicator of severe obstructive sleep apnea

**DOI:** 10.1186/s12890-022-02146-0

**Published:** 2022-09-16

**Authors:** Jung Hwan Jo, Ji Woon Park, Ji Hee Jang, Jin Woo Chung

**Affiliations:** 1 Department of Oral Medicine, Seoul National University Dental Hospital, 101 Daehak-ro, Jongno-gu, Seoul, 03080 Korea; 2 Department of Oral Medicine and Oral Diagnosis, School of Dentistry and Dental Research Institute, Seoul National University, 101 Daehak-ro, Jongno-gu, Seoul, 03080 Korea

**Keywords:** Obstructive sleep apnea, Hyoid bone, Lateral cephalography, Polysomnography, Mandibular plane, Diagnosis

## Abstract

**Background:**

The objective of this study was to evaluate the relationship between hyoid bone position and severity of obstructive sleep apnea (OSA), and to investigate its value as a complementary diagnostic method.

**Methods:**

A total of 133 patients who were diagnosed as OSA with an apnea-hypopnea index ≥ 5 were included. Clinical examination, level I polysomnography (PSG) and lateral cephalographic analysis were done. Comprehensive PSG characteristics were compared according to hyoid bone position and the predictive power of the distance between the mandible and hyoid was assessed.

**Results:**

The distance between the hyoid bone and mandibular plane was significantly longer in the severe OSA group (*p* = 0.013). The distance from hyoid bone to third vertebrae (C3) and hyoid bone to mentum were also longer in the severe OSA group but the difference did not reach statistical significance. The distance between hyoid bone and mandibular plane was effective in predicting severe OSA, with a cut-off value of 19.45 mm (AUC = 0.623, *p* = 0.040). When grouped according to a distance cut-off value of 19.45 mm, those with a longer distance between the hyoid bone and mandibular plane showed more respiratory disturbance, lower oxygen saturation levels, less deep slow wave sleep, and more fragmented sleep with arousals.

**Conclusions:**

The distance between the hyoid bone and mandibular plane derived from cephalometric analysis can be a valuable diagnostic parameter that can be easily applied in differentiating severe OSA patients.

## Background

Obstructive sleep apnea (OSA) is a disease accompanied by repeated airway obstruction during sleep. It has been known that anatomical abnormalities as well as non-anatomical factors, such as impaired muscle responsiveness, unstable respiratory control, and low respiratory arousal threshold are risk factors for OSA [1]. Another recent study revealed heritability of OSA severity between 69 and 83% [2]. OSA patients are exposed to intermittent hypoxia, leading to poor sleep quality and increased risk for various systemic diseases [3]. Therefore, its timely diagnosis and treatment is essential for general health promotion. The gold standard for OSA diagnosis is standard polysomnography (PSG) [4]. However, it cannot provide anatomical evaluation of the upper airway, limiting the identification of the obstruction site and hence predicting treatment outcomes both clinical and surgical. Anatomical parameters including airway width and length, lateral wall thickness, hyoid bone position, tongue volume and other craniofacial structures are determinate factors in the pathogenesis of certain OSA cases, so complementary diagnostic tools may be required for a comprehensive evaluation of these factors [5].

There have been numerous attempts to evaluate the upper airway anatomy in OSA patients. Imaging including videofluoroscopy, cephalometry, computed tomography (CT), and dynamic sleep magnetic resonance imaging (MRI) have been identified to play important roles in locating obstruction sites and abnormalities of hard and soft tissues of the upper airway [6–8]. Previous studies showed that certain structural craniofacial characteristics of patients observed on lateral cephalometric analysis were closely associated with pathophysiologic aspects of OSA [9, 10].

Lateral cephalography cannot be a substitute for PSG, but rather a useful supplementary diagnostic tool for OSA diagnosis. Although it captures a two-dimension view in an awake and upright condition, it is still a widely accepted clinical tool for evaluating upper airway structures with convenience at a relatively low cost. Previous studies using lateral cephalography for OSA revealed a significant correlation between OSA severity and craniofacial structures in children and adults [10, 11]. A recent meta-analysis found 646 studies on lateral cephalography for craniofacial analyses in OSA patients and revealed strong evidence for reduced pharyngeal airway space, inferiorly placed hyoid bone and increased anterior facial heights in adult OSA patients compared to controls [12]. In addition, accumulated research have supported its use as an acceptable diagnostic method that provides understanding of the anatomical etiology of OSA [13, 14].

Among the various cephalometric landmarks, indicators related to the hyoid bone have shown consistent relationships in OSA as patients having a lower hyoid bone position compared to healthy subjects [15, 16]. The hyoid bone is located within the soft tissue surrounding the pharyngeal airway and acts as an anchor to tongue muscle. So, its position plays an important role in the pathophysiology of pharyngeal airway obstruction through muscle force vectors which could be associated with increased upper airway collapsibility [17]. A recent study has suggested volumetric differences of the hyoid bone as a potential biological marker for OSA [18]. The hyoid bone is relatively easy to identify on radiographs compared to other landmarks reducing the influence of an examiner’s aptitude [16]. Although collective literature suggests hyoid bone position as a highly applicable parameter for OSA diagnosis, only a few studies specifically investigate the correlation between hyoid bone related landmarks on lateral cephalography and comprehensive PSG measurements of respiratory disturbance, sleep architecture, and oxygen saturation levels that truly reflect the disease burden of an OSA patient.

Therefore, this study aimed to evaluate the relationship among various cephalometric measurements related to hyoid bone position and objective PSG findings in OSA patients while considering the effect of potential confounders. A clinical measurement value of hyoid bone position that could be directly applied in differentiating severe OSA patients was also assessed for its predictive value.

## Methods

### Subjects

The study included 133 consecutive patients aged 20 years or older who visited Seoul National University Dental Hospital complaining of symptoms related to snoring and sleep apnea from July, 2007 to April, 2016. Medical history was collected from all patients. Physical and radiographic examinations along with level 1 nocturnal PSG were conducted. Those with an apnea-hypopnea index (AHI) ≥ 5 were included. Exclusion criteria included patients with central sleep apnea, other primary sleep disorders, history of head and neck trauma or major surgery in the orofacial region, uncontrolled cardiovascular disease or psychiatric disease, pregnancy, and medication usage for sleep disorders. This study was conducted in accordance with the amended Declaration of Helsinki. The study was approved by the Institutional Review Board of Seoul National University Dental Hospital and informed consent was obtained from all individual participants included in the study (CRI14037 and CRI20004).

### Polysomnographic evaluation

Full-night multi-channel level I PSG (Alice 5, Respironics, Pittsburgh, USA) was performed in the same clinic and scored by an sleep specialist following the standard criteria of the American Academy of Sleep Medicine [19].

Epworth Sleepiness Scale (ESS) was used to evaluate daytime sleepiness with a cut-off value of > 10 for excessive daytime sleepiness [20]. Body mass index (BMI) was based on body weight and height. Neck circumference was measured at the level of the cricothyroid membrane in the upright position.

### Cephalometric analysis

**Fig. 1:**
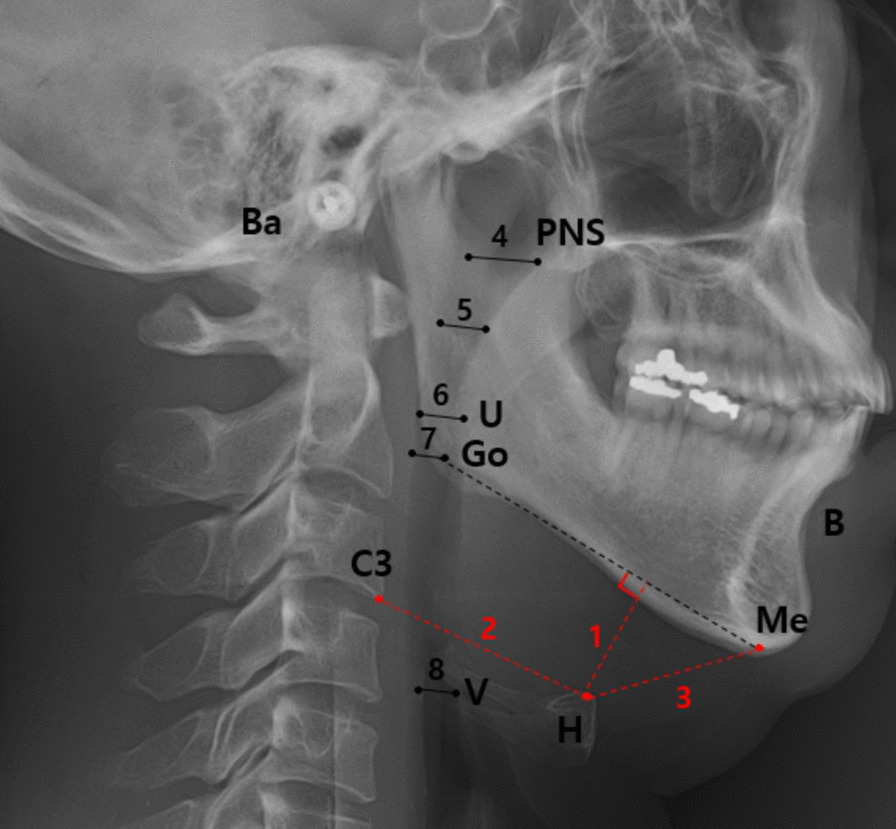
Cephalometric landmarks and linear measurements H, most anterior and superior point of hyoid bone; Go, gonion; Me, menton; B, supramentale; C3, most anterior and inferior point on the corpus to the third vertebra; PNS, posterior nasal spine; U, tip of the uvula; Ba, basion; V, vallecula; MP, mandibular plane; 1, MPH distance, perpendicular distance from H to MP; 2, H-C3, distance from H to C3; 3, H-Me: distance from H to Me; 4, NAS, nasal airway space, distance from the intersection of Ba-PNS and posterior pharyngeal wall to PNS; 5, SPAS, superior pharyngeal airway space, distance from the ventral surface of the soft palate to posterior pharyngeal wall, measured through a point midway between PNS and U along parallel to the line to Go-B plane; 6, MPAS, middle pharyngeal airway space, distance from U to posterior pharyngeal wall along parallel to the line to Go-B plane; 7, IPAS, inferior pharyngeal airway space, distance from the anterior to posterior pharyngeal wall along Go-B line; 8, HAS, hyoid airway space, perpendicular distance from V to posterior pharyngeal wall

The standardized lateral cephalograms were obtained using an Asahi CX-90 SP II (Asahi, Toshiba, Japan) and 10 × 12 inch FCR IP cassette (Fujifilm, Tokyo, Japan). The distance from the anode to midsagittal plane of the patient was 150 cm, while the distance from the midsagittal plane to IP cassette was 15 cm. Magnification factors of the images from the X-ray machine were corrected. Radiographs were taken with the subjects standing, the head fixed with ear rods and a support on the forehead, the teeth in centric occlusion position, lips in a relaxed position, and head in natural position with the sagittal plane parallel to the film at the end of expiration without swallowing. An experienced clinician who was blinded to PSG and clinical examination results performed cephalometric tracings using the digitalized V-ceph program version 5.3 (Osstem Inc., Seoul, Korea). The landmarks and linear measurements were recorded based on previous literature as shown in Fig. 1 [21, 22]: (1) H, most anterior and superior point of hyoid bone; (2) Go, gonion; (3) Me, menton; (4) B, supramentale; (5) C3, most anterior and inferior point on the corpus to the third vertebra; (6) PNS, posterior nasal spine; (7) U, tip of the uvula; (8) Ba, basion; (9) V, vallecula; (10) MP, mandibular plane; (11) MPH distance, perpendicular distance from H to MP; (12) H-C3, distance from H to C3; (13) H-Me, distance from H to Me; (14) NAS, nasal airway space, the distance from the intersection of Ba-PNS and posterior pharyngeal wall to PNS; (15) SPAS, superior pharyngeal airway space, distance from the ventral surface of the soft palate to posterior pharyngeal wall, measured through a point midway between PNS and U along parallel to the line to Go-B plane; (16) MPAS, middle pharyngeal airway space, the distance from U to posterior pharyngeal wall along parallel to the line to Go-B plane; (17) IPAS, inferior pharyngeal airway space, distance from the anterior to posterior pharyngeal wall along Go-B line; (18) HAS, hyoid airway space, perpendicular distance from V to posterior pharyngeal wall.

### Statistical analysis

Differences in demographic, clinical and hyoid bone position indices according to OSA severity were analyzed by independent t-test, chi-square test and Fisher’s exact test. Logistic regression analyses were conducted to determine clinical and hyoid bone position indices predicting severe OSA. The receiver operating characteristic (ROC) curve and area under the curve (AUC) were analyzed to obtain the cut-off value for age, BMI, ESS and distance from hyoid bone to mandibular plane in evaluating severe OSA. Differences in polysomnographic indices according to age, BMI, and hyoid bone position using a cut-off value were analyzed by independent t-test and chi-square test. SPSS 25.0 software (IBM, Chicago, IL, USA) was used for all statistical analysis. The level of statistical significance was set at *p* < 0.05.

## Results

### Demographic characteristics

Of the total 133 patients evaluated there were 113 men (85.0%) and 20 women (15.0%) with a mean age of 44.0 ± 12.3 years (range, 20–82 years). Among them, 86 patients had mild to moderate OSA (mean age 41.5 ± 12.9 years) and 47 patients had severe OSA (mean age 48.4 ± 10.0 years).

As shown in Table 1, patients with severe OSA were older (*p* = 0.002) and showed higher BMI (*p* < 0.001), neck circumference (*p* = 0.030), and comorbid hypertension (*p* = 0.004) compared to patients with mild to moderate OSA. Patients with severe OSA showed higher male ratio, ESS score, and rate of comorbid diabetes mellitus compared to patients with mild to moderate OSA, but the difference was not statistically significant.

**Table 1:** Clinical and hyoid bone position indices according to obstructive sleep apnea severity

Variable	Mild to moderate OSA (n = 86)	Severe OSA (n = 47)	*p*-value
Age (years)^a^	41.5 (12.9)	48.4 (10.0)	0.002**
Age > 55 years^b^	12.8%	27.7%	0.033*
Male^b^	81.4%	91.5%	0.120
BMI (kg/m^2^)^a^	24.5 (3.3)	26.7 (3.1)	< 0.001**
Neck circumference (cm)^a^	36.0 (5.8)	38.7 (6.8)	0.030*
ESS^a^	7.8 (3.7)	9.1 (5.2)	0.111
ESS > 10^b^	22.2%	25.5%	0.670
Hypertension^b^	16.9%	40.9%	0.004**
Diabetes mellitus^c^	5.2%	9.1%	0.460
Hyoid bone position (mm)^a^			
Hyoid to mandibular plane	19.2 (5.9)	21.9 (6.2)	0.013*
Hyoid to C3	42.4 (5.8)	43.8 (5.2)	0.296
Hyoid to mentum	42.4 (7.2)	43.4 (7.4)	0.467
NAS (mm)^a^	25.8 (3.9)	25.0 (3.1)	0.235
SPAS (mm)^a^	12.1 (3.7)	10.9 (3.0)	0.052
MPAS (mm)^a^	9.1 (3.6)	9.0 (3.2)	0.821
IPAS (mm)^a^	11.5 (4.0)	10.1 (3.2)	0.035*
HAS (mm)^a^	18.3 (5.5)	17.5 (5.7)	0.430

*OSA* Obstructive sleep apnea; *BMI* Body mass index; *ESS* Epworth sleepiness scale; *C3* The third vertebra; *NAS* Nasal airway space; *SPAS* Superior pharygeal airway space; *MPAS* Middle pharygeal airway space; *IPAS* Inferior pharygeal airway space; *HAS* Hyoid airway space

^a^Results were obtained from independent t-test: mean (SD)

^b^Results were obtained from chi-square test

^c^Results were obtained from Fisher’s exact test

*Significant difference: *p* < 0.05

**Significant difference: *p* < 0.01

### Differences in hyoid bone position according to OSA severity

Cephalometric analysis showed that the distance between the hyoid bone and mandibular plane was significantly longer in the severe OSA group (*p* = 0.013). The distance from hyoid bone to third vertebrae (C3) and hyoid bone to mentum were also longer in the severe OSA group but the difference did not reach statistical significance. The severe OSA group showed a narrower airway space at all levels compared to the mild to moderate OSA group. However, the difference was statistically significant only for the inferior pharyngeal airway space (*p* = 0.035).

### Effectiveness of clinical and hyoid bone position indices in predicting severe OSA

Table 2 shows clinical and hyoid bone position variables associated with severe OSA based on logistic regression analysis. The distance between the hyoid bone and mandibular plane (β = 0.075, *p* = 0.039) was a significant predictor of severe OSA after adjusting for age, gender and BMI.

**Table 2:** Clinical and hyoid bone position indices predicting severe obstructive sleep apnea

Predictor variables	Standardized β	Standard error	Odds ratio	95% CI	*p*-value
Age (years)	0.058	0.021	1.060	1.017–1.105	0.005**
Gender (Male)	2.020	0.859	7.536	1.400–40.570	0.019*
BMI (kg/m^2^)	0.361	0.108	1.434	1.160–1.774	0.001**
Neck circumference (cm)	0.039	0.037	1.040	0.967–1.119	0.293
ESS	0.135	0.062	1.145	1.015–1.292	0.028*
Distance from hyoid to mandibular plane (mm)	0.075	0.044	1.077	0.989–1.174	0.039*
Distance from hyoid to C3 (mm)	− 0.104	0.059	0.902	0.803–1.012	0.079
Distance from hyoid to mentum (mm)	− 0.019	0.039	0.981	0.908–1.060	0.627

*CI* Confidence interval; *BMI* Body mass index; *ESS* Epworth sleepiness scale; *C3* The third vertebra

Results were obtained from logistic regression analysis

*Significant difference: *p* < 0.05

**Significant difference: *p* < 0.01

**Fig. 2:**
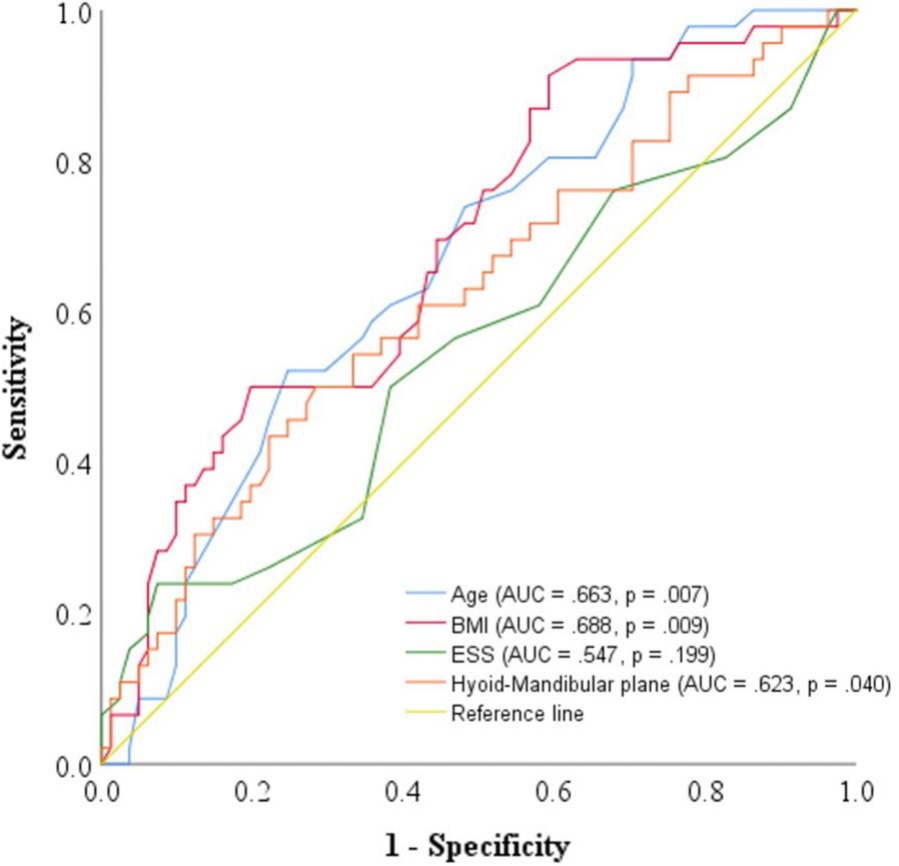
Receiver operating characteristic (ROC) curve analysis for clinical and hyoid bone position indices in predicting severe obstructive sleep apnea. *BMI* Body mass index; *ESS* Epworth sleepiness scale; *AUC* Area under the curve

As shown in Fig. 2 and Table 3, the distance between the hyoid bone and mandibular plane was effective in predicting sever OSA, along with age and BMI. The ROC curve analysis the distance between the hyoid bone and mandibular plane with a cut-off value of 19.45 mm led to an AUC of 0.623 (*p* = 0.040). Age with a cut-off value of 45 years led to an AUC of 0.663 (*p* = 0.007) and BMI with a cut-off value of 25 kg/m^2^ led to an AUC of 0.688 (*p* = 0.009).

**Table 3:** Sensitivity, specificity, PPV, NPV, and error rate of clinical and hyoid bone position indices in evaluating severe obstructive sleep apnea

Cut-off value		OSA group		AUC	Sensitivity (%) [95% CI]	Specificity (%) [95% CI]	PPV (%) [95% CI]	NPV (%) [95% CI]	Error rate (%)	*p*-value
		Severe	Mild to moderate							
Distance from hyoid bone to mandibular plane	≥ 19.45 mm	29	38	0.632	61.7 [46.4, 75.5]	55.8 [44.7, 66.5]	43.3 [35.5, 51.4]	72.7 [63.9, 80.1]	42.1	0.040*
	< 19.45 mm	18	48							
Age	≥ 45 years	29	32	0.663	61.7 [46.4, 75.5]	62.8 [51.7, 73.0]	47.5 [38.9, 56.4]	75.0 [66.8, 81.7]	37.6	0.007**
	< 45 years	18	54							
BMI	≥ 25 kg/m^2^	32	39	0.688	69.6 [54.3, 82.3]	54.1 [43.0, 65.0]	45.1 [37.8, 52.6]	76.7 [67.1, 84.1]	40.5	0.009**
	< 25 kg/m^2^	14	46							
ESS	≥ 9	23	31	0.547	50.0 [34.9, 65.1]	61.7 [50.3, 72.3]	42.6 [33.2, 52.5]	68.5 [60.8, 75.3]	42.5	0.199
	< 9	23	50							

Sensitivity was obtained from TP/(TP + FN) × 100, Specificity was obtained from TN/(TN + FP) × 100, PPV was obtained from TP/(TP + FP) × 100, NPV was obtained from TN/(TN + FN) × 100, Error rate was obtained from (FN + FP)/(TN + TP + FN + FP)

*OSA* Obstructive sleep apnea; *AUC* Area under the curve; *CI* Confidence internal; *BMI* Body mass index; *ESS* Epworth sleepiness scale

*Significant difference: *p* < 0.05

**Significant difference: *p* < 0.01

### Relationship between clinical and polysomnographic indices and the distance between the hyoid bone and mandibular plane (MPH distance)

As shown in Table 4, the longer MPH distance was significantly correlated with higher total AHI (r = 0.318, *p* = 0.001), supine AHI (r = 0.313, *p* = 0.014), non-supine AHI (r = 0.312, *p* = 0.001), non-rapid eye movement (NREM) AHI (r = 0.338, *p* = 0.001), percentage of time below 90% SpO_2_ (r = 0.327, *p* = 0.001), NREM arousal index (r = 0.276, *p* = 0.002), respiratory arousal index (r = 0.329, *p* = 0.001), total arousal index (r = 0.323, *p* = 0.001), percentage of stage I sleep (r = 0.276, *p* = 0.001) and lower mean SpO_2_ (r = − 0.263, *p* = 0.003), lowest SpO_2_ (r = − 0.208, *p* = 0.017), NREM SpO_2_ (r = − 0.268, *p* = 0.002), and percentage of sleep stage II (r = − 0.208, *p* = 0.016).

**Table 4:** Correlation between clinical and polysomnographic variables and hyoid bone position

Predictor variables	r	*p*-value
Age (years)	0.096	0.270
BMI	0.158	0.072
Neck circumference (cm)	0.166	0.089
Total AHI	0.318	0.001**
Supine AHI	0.313	0.001**
Non-supine AHI	0.312	0.001**
REM AHI	0.141	0.107
NREM AHI	0.338	0.001**
Mean SpO_2_	− 0.263	0.003**
Lowest SpO_2_	− 0.208	0.017*
Time below 90% SpO_2_ (%)	0.327	0.001**
REM SpO_2_	0.002	0.981
NREM SpO_2_	− 0.268	0.002**
REM arousal index	0.167	0.064
NREM arousal index	0.276	0.002**
Respiratory arousal index	0.329	0.001**
Total arousal index	0.323	0.001**
Total sleep time (mins)	− 0.145	0.095
Sleep efficiency (%TST)	− 0.163	0.061
Sleep latency (mins)	− 0.018	0.833
REM latency (mins)	− 0.037	0.674
Sleep stage I (%TST)	0.276	0.001**
Sleep stage II (%TST)	− 0.208	0.016*
Sleep stage III + IV (%TST)	− 0.094	0.282
REM sleep (%TST)	− 0.020	0.819
Time of supine position (% TST)	− 0.089	0.309

Results were obtained from Pearson correlation analysis

*BMI* Body mass index; *AHI* Apnea-hypopnea index; *REM* Rapid eye movement sleep; *NREM* Non-rapid eye movement sleep

*Significant difference: *p* < 0.05

**Significant difference: *p* < 0.01

### Differences of clinical and polysomnographic indices according to MPH distance

When the patients were divided according to MPH distance at a cut-off value of 19.45 mm, the longer MPH distance group showed a higher male ratio (*p* = 0.048) and percentage of those with a ESS score > 10 (*p* = 0.047) compared to the shorter MPH distance group.

Table 5 shows polysomnographic values according to MPH distance. The longer MPH distance group showed significantly higher total AHI (*p* = 0.002), supine AHI (*p* = 0.002), non-supine AHI (*p* = 0.004), NREM AHI (*p* = 0.001), percentage of severe OSA (*p* = 0.040) and lower mean SpO_2_ (*p* = 0.013), NREM SpO_2_ (*p* = 0.009), higher percentage of time below 90% SpO_2_ (*p* = 0.003) and percentage of stage I sleep (*p* = 0.006), lower percentage of sleep stage II (*p* = 0.026), higher NREM arousal index (*p* = 0.016), respiratory arousal index (*p* = 0.001), and total arousal index (*p* = 0.001) compared to the shorter MPH distance group.

**Table 5:** Polysomnographic indices according to hyoid bone position

Variable	Longer MPH distance group (n = 67)	Shorter MPH distance group (n = 66)	*p*-value
Total AHI^a^	33.9 (27.1)	21.8 (16.2)	0.002**
Supine AHI^a^	42.3 (29.7)	28.1 (20.7)	0.002**
Non-supine AHI^a^	16.4 (25.6)	6.3 (11.3)	0.004**
REM AHI^a^	36.6 (25.6)	33.4 (20.8)	0.428
NREM AHI^a^	33.0 (28.5)	19.4 (17.2)	0.001**
Severe OSA group^b^	43.3%	27.3%	0.040*
Mean SpO_2_ (%)^a^	94.7 (2.1)	95.6 (1.6)	0.013*
Lowest SpO_2_ (%)^a^	79.1 (10.1)	82.0 (9.0)	0.084
REM SpO_2_ (%)^a^	94.1 (3.3)	93.9 (11.9)	0.890
NREM SpO_2_ (%)^a^	94.7 (2.2)	95.6 (1.6)	0.009**
Time below 90% SpO_2_ (%)^a^	7.4 (11.2)	2.7 (4.6)	0.003**
Total sleep time (min)^a^	315.3 (66.8)	326.3 (60.9)	0.326
Sleep efficiency (%)^a^	79.0 (13.0)	82.2 (10.3)	0.121
Sleep latency (min)^a^	17.1 (17.8)	15.1 (15.0)	0.494
REM latency (min)^a^	113.1 (54.5)	125.1 (62.7)	0.240
Sleep stage I (%)^a^	31.7 (17.0)	24.4 (12.9)	0.006**
Sleep stage II (%)^a^	45.4 (14.7)	50.9 (13.4)	0.026*
Sleep stage III + IV (%)^a^	1.8 (3.8)	3.2 (6.5)	0.121
REM sleep (%)^a^	16.9 (7.0)	16.8 (6.9)	0.931
Time of supine position (%)^a^	68.7 (26.9)	76.2 (21.9)	0.078
REM arousal index^a^	18.5 (15.7)	15.2 (13.1)	0.211
NREM arousal index^a^	18.7 (19.0)	11.4 (13.3)	0.016*
Respiratory arousal index^a^	22.6 (20.7)	12.7 (12.3)	0.001**
Total arousal index^a^	34.8 (17.9)	24.8 (12.3)	0.001**

*MPH distance* The distance between hyoid bone and mandibular plane; *AHI* Apnea-hypopnea index; *REM* Rapid eye movement sleep; *NREM* Non-rapid eye movement sleep; *OSA* Obstructive sleep apnea; *SpO*_2_ Oxygen saturation; *TST* Total sleep time

^a^Results were obtained from independent t-test: mean (SD)

^b^Results were obtained from chi-square test

*Significant difference: *p* < 0.05

**Significant difference: *p* < 0.01

## Discussion

The results of this study showed that the distance between the hyoid bone and mandibular plane was significantly longer in the severe OSA group. When grouped according to a distance cut-off value of 19.45 mm suggested from the results, those with a longer MPH distance showed more respiratory disturbance, lower oxygen saturation levels, less deep slow wave sleep, and more fragmented sleep with arousals.

Anatomical features of the upper airway including tongue size and position and pharyngeal length and cross-sectional area play a major role in the etiology of OSA. Muscles that maintain upper airway patency including the middle pharyngeal constrictor muscle attach to the hyoid bone and many muscles such as intrinsic tongue muscles, genioglossus, and geniohyoid muscle that are involved in tongue movement and position. Hence, upper airway configuration, pharyngeal patency, and tongue movement are all related to the position of the hyoid bone [23]. Pharyngeal length is the distance between the posterior nasal spine and base of the epiglottis that is attached to the hyoid bone [24]. A longer pharyngeal length has been generally associated with higher pharyngeal collapsibility and a higher AHI value [25, 26]. Additionally, larger tongue dimensions have been associated with an inferiorly displace hyoid bone [27]. On the other hand, studies showed that treatment with oral appliances that position the mandible forwardly resulted in an elevation of hyoid bone position as much as 9 mm based on the distance from the inferior border of the mandible [28] and isolated hyoid surgery resulted in a 38% mean reduction in AHI in OSA patients [29].

As shown by the results of this study, severe OSA patients with an AHI value ≥ 30 showed a significantly lower hyoid bone position expressed as a longer distance from the mandibular plane with a mean value of 22 mm. This is approximately 2.5 mm longer distance compared to the normative value of 19.5 mm suggested for healthy men [30]. Pharyngeal length is generally known to be longer in males compared to females and this has been suggested as a contributing factor to the sex differences in upper airway mechanism and OSA prevalence [31]. Such results are in line with our study showing a significant higher male ratio in the low hyoid bone position group. Overall the results of this study support previous literature suggesting the close association between pharyngeal length and upper airway collapsibility, and the importance of anatomical factors in severe OSA.

The distance between the hyoid bone and mandibular plane is the most commonly evaluated parameter in cephalography based studies although it could not as itself be a sufficient tool to predict treatment outcomes. Most of the literature shows that among numerous cephalometric variables the distance between these two is the only parameter that shows a significant influence on AHI difference [10] and consistently differentiates OSA patients from healthy controls [15]. And it has been found that lower hyoid bone position was related to poor treatment prognosis in OSA [32, 33]. The distance between the hyoid bone and mandibular plane range from 22 to 26 mm according to the study and the value of 22 mm from our study is also in accordance with such results [15].

The value of the distance between the hyoid and mandibular plane as a diagnostic index for severe OSA was shown in the results of regression and ROC analysis of this study. The value was significantly associated with severe OSA along with well-established risk factors such as age and BMI [6]. A cut-off value that can be directly applied in predicting severe OAS patients before standard diagnostic procedures was suggested as 19.45 mm. Although not many studies provide such data, a previous study suggested 18 mm as the distance between the hyoid bone and mandibular plane that indicated an increased risk of having and AHI ≥ 15 [34]. The difference in the cut-off value from the two studies may suggest an incremental change in such values according to OSA severity group.

Previous studies investigating the hyoid related cephalometric variables in OSA patients are often limited to analyzing their relationship with respiratory disturbance values such as AHI [33, 35]. In our study the investigation was expanded to include a wider range of variables related to oxygen saturation and sleep architecture to provide a more comprehensive view of the differences in sleep according to hyoid bone position. Those with a longer MPH distance showed a significantly higher AHI in both supine and non-supine position implicating that hyoid bone position may have an effect on respiratory disturbance level regardless of sleep position. Interestingly REM AHI did not significantly differ according to hyoid bone position group. This could reflect a sleep stage dependency of anatomical influence. OSA worsens during REM sleep whereas REM OSA may be an independent risk factor for adverse health outcomes related to OSA [36]. Muscle atonia is characteristic of REM sleep and the extent of upper airway muscle relaxation and loss of responsiveness may be irrelevant to pharyngeal length during this stage. The worsened oxygen saturation indices in the low hyoid bone position group showing that respiratory disturbance was leading to intermittent hypoxia, which could be more directly related to adverse systemic sequelae and increased mortality of OSA [37]. OSA patients with a longer MPH distance were also accompanied by more arousals and less restorative slow wave sleep. The above results show that patient grouping based on the distance between hyoid bone and mandibular plane was sufficient to differ OSA patients of different severity not only in aspects of respiratory disturbance but also in hypoxic burden and sleep fragmentation which are the hallmarks of OSA. The cut-off value deduced from this study is more reliable considering the fact that differences in various PSG variables were significant while both groups according to hyoid position did not show a significant difference in well-known confounders of OSA severity including age and BMI.

Certain orthopedic factors may affect hyoid bone position. Changing natural head posture to 20 degrees extension produced changes in lordosis which showed significant correlations with hyoid bone position and free airway size [38]. Curvature of the cervical spine (lordosis, straight, or kyphosis) was found to be more closely related to hyoid bone position than the craniocervical relationship [39]. Furthermore, the distance between the hyoid bone and mandibular plane was significantly larger in upright compared to supine position supporting a postural effect [40, 41].

There are several limitations of this study that should be considered in interpreting the results. First, the study lacks a healthy control group that could add value in further comparisons among groups. Also the study is of a retrospective nature and other confounders of OSA may not have been properly controlled. Future prospective studies should be designed with OSA patients of a wider range of severity while considering more confounders such as alcohol intake, smoking, and physical activity level to clarify the true causal relationship between hyoid bone position and OSA severity. Second, the cross-sectional design of this study limits the possibility of establishing causality between hyoid bone position and OSA. Further studies are necessary to elucidate the longitudinal effect of OSA on craniofacial structures. Third, the cut-off value introduced in this study should be tested in diverse patient populations to verify its reliability in further studies. However, this study involved OSA patient grouping based on objective level I PSG data obtained from a single test center with regular concordance activities and standardized protocols, assigning reliability to the derived result. Also the relatively large sample size and comprehensive analysis of PSG variables compared to other studies of similar study design is an advantage of this study. Finally, lateral cephalography itself has its limitations as a two-dimensional sagittal image taken while awake in a sitting position, so no information is provided on the medio-lateral pharyngeal dimensions [13, 14]. In spite of the reported shortcomings of cephalography, its appeal as a relatively convenient, simple and low-cost diagnostic tool for both clinician and patient is undeniable. Future studies based on functional imaging methods including dynamic MRI and computational fluid dynamics with CT would be necessary to better characterize the role of hyoid bone position, surrounding muscles, and airway obstruction under three-dimensional dynamic conditions. Also, this would help to improve knowledge of functional anatomy and the true diagnostic role of hyoid bone position in OSA.

## Conclusions

The results of this study support the utility of the distance between the hyoid bone and mandibular plane derived from cephalometric analysis as a complementary diagnostic parameter that has sufficient power in differentiating severe OSA patients based on extensive standard PSG results. The hyoid bone to mandibular plane distance provided in this study could be directly applied in preliminary screening and treatment planning for OSA patients.

## Data Availability

The datasets used and/or analyzed during the current study are available from the corresponding author on reasonable request.

## Abbreviations

OSA: Obstructive sleep apnea
PSG: Polysomnography
AHI: Apnea-hypopnea index
ESS: Epworth sleepiness scale
BMI: Body mass index
REM: Rapid eye movement
NREM: Non-rapid eye movement
C3: The third vertebrae
